# Acute and Severe Hypercalcemia Early After Kidney Transplantation in a Patient Previously Treated With Etelcalcetide

**DOI:** 10.3389/ti.2023.11271

**Published:** 2023-06-13

**Authors:** Maxime Foguenne, Michel Mourad, Antoine Buemi, Tom Darius, Nada Kanaan, Michel Jadoul, Laura Labriola, Arnaud Devresse

**Affiliations:** ^1^ Department of Abdominal Surgery and Transplantation, Cliniques Universitaires Saint-Luc, Université catholique de Louvain, Brussels, Belgium; ^2^ Department of Nephrology, Cliniques Universitaires Saint-Luc, Université catholique de Louvain, Brussels, Belgium

**Keywords:** kidney transplant, calcium-sensing receptor (CASR), hyperparathyroidism, kidney transplant clinics, parathyroid

Dear Editors,

Secondary hyperparathyroidism (SHPT) is frequent in patients with chronic kidney disease, especially in those on chronic haemodialysis (HD). Etelcalcetide, an intravenously-administered direct CaSR-agonist, is widely used worldwide for SHPT treatment. Yet, little has been described so far regarding its potential post-kidney transplant (KT) impact. We previously reported acute and severe hypercalcemia in the early post-transplant course in two patients previously treated with high-dose etelcalcetide [1]. We here report another case.

A 68-year-old Caucasian male received a deceased-donor KT for kidney failure of unknown origin. He has been on HD for 4 years and treated with vitamin D analogue and etelcalcetide (15 mg/dialysis session, last dose the day before KT) for 2 years for SHPT. Pre-transplant serum calcium and iPTH values-measured the day before transplantation-were 2.30 mmol/L (2.15–2.50 mmol/L) and 50.9 pmol/L (1.6–8.5 pmol/L), respectively.

The first week post-KT was uncomplicated. Kidney function rapidly improved and calcemia remained within the normal range. On day 8, the patient presented tonic-clonic seizures associated with severe hypertension. Brain MRI was suggestive for PRES-syndrome. Laboratory tests revealed severe hypercalcemia (total serum calcium 3.25 mmol/L, contrasting with a normal value 3 days before), hypophosphatemia (0.74 mmol/L, [0.81–1.45 mmol/L]), and elevated iPTH level at 65.6 pmol/L (Figure 1). Tacrolimus trough level at 30 ng/mL while two previous dosages (on day 3 and day 5) were into targets (10–14 ng/mL) with an unchanged dose at 25 mg/day. Hematologic and auto-immune tests were normal and pre-KT radiologic findings showed no bone lesion.

**FIGURE 1:**
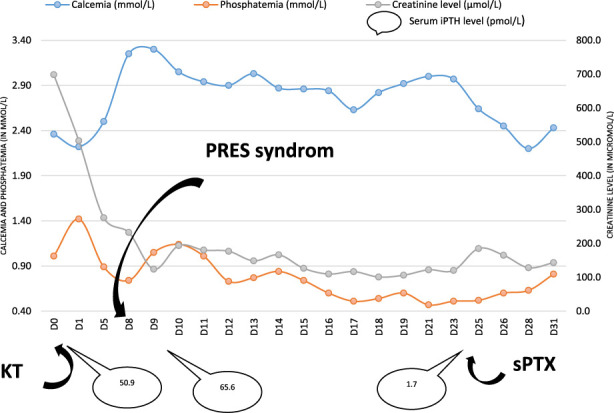
Calcemia, phosphatemia, creatininemia and iPTH serum level evolution. Abbreviations: KT: kidney transplantation; sPTX: subtotal parathyroidectomy.

Antiepileptic drug (levetiracetam 2.000 mg/day), cinacalcet (120 mg/day), anti-hypertensive treatment, intravenous hydration and tacrolimus posology reduction were initiated. Cervical MRI showed two parathyroid hyperplasia’s foci.

Two weeks later, the neurological status of the patient improved. Kidney function continued to improve with plasma creatinine values around 106 μmol/L (53–115 μmol/L). Yet, calcemia remained constantly >2.90 mmol/L despite cinacalcet (that was poorly tolerated, causing nausea and vomiting). Therefore, we performed on day 24 a subtotal parathyroidectomy by resecting *in toto* the two parathyroid hyperplastic foci and the left superior parathyroid gland, together with a partial resection of the right inferior one. Pathological examination confirmed the diagnosis of tertiary hyperparathyroidism.

After surgery, calcium, phosphate and iPTH values returned into the normal range (Figure 1) and clinical symptoms resolved rapidly. A brain MRI was repeated on day 20 and was normal. The patient was discharged on day 36.

Overall, we report another case of severe and acute hypercalcemia occurring early after KT, most likely related to SHPT flare-up secondary to etelcalcetide interruption, that prompted early parathyroid surgery. In our knowledge, such severe clinical presentation has not been reported before the etelcalcetide era, even in patients treated with cinacalcet. Indeed, although pre-transplant cinacalcet treatment has been shown to potentially induce hyperparathyroidism rebound, nephrocalcinosis and secondary hypercalcemia developing usually months after KT [2, 3], hypercalcemia usually does not exceed 2.9 mmol/L and rarely requires any acute treatment-contrasting with the clinical presentation of the present case and those previously published [1].

Also the causal relationship between acute hypercalcemia and PRES-syndrome cannot be definitively proven here-as the patient presented with concurrent severe hypertension and tacrolimus overdose-it might have participated in this severe manifestation [4, 5].

In conclusion, patients treated with high-dose of etelcalcetide require close monitoring of calcium levels after transplantation. Larger studies are required to confirm our observation and assess the causal relationship between etelcalcetide and severe post KT hypercalcemia.

## Data Availability

The raw data supporting the conclusion of this article will be made available by the authors, without undue reservation.
